# Objective assessment of urban built environment related to physical activity — development, reliability and validity of the China Urban Built Environment Scan Tool (CUBEST)

**DOI:** 10.1186/1471-2458-14-109

**Published:** 2014-02-04

**Authors:** Meng Su, Yu-kun Du, Qing-min Liu, Yan-jun Ren, Ichiro Kawachi, Jun Lv, Li-ming Li

**Affiliations:** 1 Department of Epidemiology and Biostatistics, School of Public Health, Peking University, 38 XueYuan Road, HaiDian District, Beijing 100191, China; 2 Department of Chronic Non-communicable Disease Control and Prevention, Hangzhou Center for Disease Control and Prevention, Hangzhou 310021, China; 3 Department of Social and Behavioral Sciences, School of Public Health, Harvard University, Boston, MA 02115, USA

**Keywords:** Environmental scan, Physical activity, Reliability, Validity

## Abstract

**Background:**

Some aspects of the neighborhood built environment may influence residents’ physical activity, which in turn, affects their health. This study aimed to develop an urban built environment evaluation tool and conduct necessary reliability and validity tests.

**Methods:**

A 41-item urban built environment scan tool was developed to objectively assess the neighborhood built environment features related to physical activity. Six neighborhoods in Hangzhou were selected from three types of administrative planning units. A pair of auditors independently assessed all of the 205 street segments at the same time. Half of the segments (n = 104) were audited twice by the same auditor after a two-week time interval. Inter-rater reliability was assessed by comparing the audits of paired observers, while intra-rater reliability was evaluated by comparing an auditor’s repeated assessments of the same segments. The construct validity was tested using factor analysis.

**Results:**

The inter-rater reliability for most items was above 0.8. The intra-rater reliability for most items was above 0.4, and was lower than corresponding inter-rater reliability. Six factors were extracted by factor analysis and the factor loading matrix showed good construct validity.

**Conclusions:**

The CUBEST is a reliable and valid instrument that can be used to assess the physical activity-related built environment in Hangzhou, and potentially other cities in China.

## Background

Physical inactivity is an important, modifiable behavioral risk factor for non-communicable chronic diseases [1]. Epidemiological studies have shown that physical inactivity is associated with increased risks of obesity, diabetes, cardiovascular disease, and other chronic diseases [2,3]. A growing number of studies have focused on the ecological context of physical activity [4], i.e. the influence of the residential built environment on physical activity patterns [5].

The built environment—the physical form of communities usually consists of 6 dimensions: (1) residential density; (2) street connectivity; (3) accessibility to destinations (land-use mixed) and services; (4) walking and cycling environment; (5) aesthetic quality; and (6) safety. Together, these elements shape access to opportunities for physical activity. Three types of data are usually used to measure built environment attributes believed to be related to physical activity: (1) perceived (subjective) measures obtained by personal interview with questionnaire; (2) observational (objective) measures obtained using systematic scans or audits; and (3) archival data sets that are often layered and analyzed with GIS [6].

China is experiencing unprecedented rates of urbanization, which have contributed to the decline of physical activity [7] and dramatically changes in built environment. For example, sprawling development, transportation infrastructures that emphasizes driving, and inconvenient, unsafe, and unpleasant public open spaces have been discouraging active transportation behaviors like walking and bicycling [8]. Thus, it is critical to accurately assess built environment characteristics before understanding how they correlate with physical activity in China. Some instruments have been developed for assessing the built environment in developed countries, yet few have been done in China [9]. Furthermore, unique built environmental features are presented in Chinese and other Asian ultra-dense cities, which could bring about different associations with physical activities. These include but not limited to mixed types of residential housing; crowdedness; a complex public transport network; a great number of overpasses; bike parking facilities; man-made obstacles on sidewalks and bike lanes; and unique types of destinations (e.g., historical buildings, tea houses, open-air food outlets). In additional, even the same feature of built environment could correlate with physical activity differently between in China and other Western countries. For example, positive relationship is usually observed between residential density and physical activity in the Western countries. But studies have suggested the possibility that densely settled Chinese cities could hinder leisure-time physical activity due to decreased availability of physical activity resources and increased concerns about traffic safety [10]. These facts show that the application of current instruments on assessing Chinese urban built environment is limited. Therefore, it is of vital importance to make necessary adjustments to have an instrument specifically designed for urban built environment in China.

In this study, we created an adapted instrument (observational measures obtained using systematic scans or audits) based on a review of existing reliable auditing tools. This instrument is specifically designed for urban built environment related to adult physical activity in mainland China, that is, the China Urban Built Environment Scan Tool (CUBEST). This article sought to report the reliability and validity assessment results of the CUBEST conducted in the city of Hangzhou, China.

## Methods

### Study site

The city of Hangzhou, which is the capital of Zhejiang Province, is situated in the southeast coastal area of China. Hangzhou exercises jurisdiction over eight districts, three county-level cities, and two counties. It is an economically developed city in China, and its comprehensive economic strength ranked eighth among all large- and medium-sized cities in China in 2011. By the end of 2011, the population of long-term residents in the city was 8.74 million, of which the urban population is 6.45 million, accounting for 73.9% [11]. Two districts located in a central geographic location of Hangzhou were included to test the reliability and validity of the CUBEST, i.e., Shangcheng District and Xiacheng District.

All administrative planning units in these two districts are classified into five categories [12] based on the degree of land-use mix and service capacity of public buildings. Public buildings usually consist of buildings used for government, commercial, educational, transport and health care purposes. A Type I unit is characterized by fully developed commercial and residential areas with dense population and mixed land use. A Type II unit has developed but scattered public buildings as a feature, lacking of comprehensive service capacity. A Type III unit is featured by partly developed and single functional public buildings. Type IV and type V units are mainly composed of farmland and industrial storage warehouses and were excluded from this study. A typical neighborhood in most urban areas of China usually shows a shape of square or rectangle with 0.2 to 0.5 square kilometers in area. In this study, we extended 400 meters out from each side of the neighborhood boundaries to form a study area with 1.0 to 1.5 square kilometers in area. Two neighborhoods were selected in each of the three types of units and all the street segments in these 6 extended study areas were evaluated using environmental audit instrument. A street segment was defined as a section of street or road between two intersections with a maximum length of 400 meters.

### Development of the CUBEST

The CUBEST was designed based on a review of existing reliable instruments which have been developed since the year 2000, including Analytic Audit Tool [13], Active Neighborhood Checklist [14], SSO [15], PIN3 Neighborhood Audit Tool [16], Irvine - Minnesota Inventory [17], NALP [18], EAST_HK [9], SPACES [19], PEDS [20], WABSA [21], Sidewalk Assessment Tool [22], and PARA [23]. Table 1 shows the information on the above-mentioned instruments assessing neighborhood built environment related to physical activity. An item pool was generated mainly from the Analytic Audit Tool, Active Neighborhood Checklist and EAST_HK after consideration of the date of development, sample size, comprehensiveness of dimensions, survey method and time cost of current instruments. Six dimensions were involved in the CUBEST, including residential density, street connectivity, accessibility (land-use mix), sidewalk quality, bike lane quality, and aesthetic. Items belonged to the dimension of safety from traffic were integrated into the dimensions of sidewalk and bike lane quality. Meanwhile, necessary adjustments were made to fit in the characteristics of Chinese urban settings. Items with Chinese and local characteristics were added, such as “Cycling or walking against the flow of traffic”, “Cultural features (historical buildings) in the neighborhood”, “Government sponsored public recreational equipments”, “Tea house”, “West Lake or Beijing-Hangzhou Grand Canal”. Physical activity facilities were categorized to 9 kinds of destinations based on the PANES questionnaire [24].

**Table 1:** Information on 12 instruments assessing neighborhood built environment related to physical activity

**Instrument**	**Number of items**	**Time required**	**Dimensions**						
			**Residential density**	**Accessibility**	**Street connectivity**	**Sidewalks**	**Bike lane**	**Aesthetic quality**	**Safety**
Analytic Audit Tool	27	10.6 min/segment	✔	✔	✔	✔	✔	✔	✔
Active Neighborhood Checklist	89	11.7 min/segment	✔	✔	✔	✔	✔	✔	✔
EAST_HK	91	12.38 min/segment	✔	✔	✔	✔		✔	✔
Irvine - Minnesota Inventory	162	20 min/segment	✔	✔	✔	✔	✔	✔	✔
Neighborhood Active Living Potential (NALP)	18	No report		✔		✔	✔		✔
Pedestrian Environment Data Scan Tool (PEDS)	36	3–5 min/400 ft. segment				✔	✔		✔
Physical Activity Resource Assessment Instrument (PARA)	43	10 min/ location		✔					
PIN3 Neighborhood Audit Instrument	43	No report	✔	✔		✔	✔	✔	✔
Sidewalk Assessment Tool	5	8–12 min/segment				✔		✔	
Systematic Pedestrian and Cycling Environment Scan (SPACES)	51	20 min/Km		✔		✔	✔	✔	✔
Systematic Social Observation (SSO)	126	5–10 min/segment	✔	✔		✔	✔	✔	
Walking/Bicycling Suitability Assessment Form (WABSA)	16 + 27	No report				✔	✔		✔

The draft of the CUBEST contained 49 items under 6 dimensions, including residential density (4 items), street connectivity (3 items), accessibility (21 items), sidewalk quality (6 items), bike lane quality (8 items), and aesthetics (7 items).

### On-site evaluation

The validation of the CUBEST was conducted in Hangzhou City from October to December in 2011. In-the-field audits of street segments was conducted by two raters who were also involved in the development of the CUBEST. A standard operating procedure for environmental audit was developed using detailed written instructions and field pictures to achieve uniformity in the performance of evaluation. A two-day intensive rater training was developed with the goal of making them become proficient at completing the measures, including explanation of the principles, operation, potential problems and solutions of the CUBEST and GPS positioning device. Before the evaluation was begun across the whole sample, two raters independently finished a pilot evaluation of two neighborhoods (about 60–80 street segments) using the CUBEST. Any discrepancies were resolved by another one of the developers of the CUBEST. In the formal survey, the two raters independently performed evaluations of a total of 205 street segments at the same time. Approximately half of the street segments (n = 104) were randomly sampled and independently scanned by the two raters for the second time after a two-week time interval. All environmental scans were conducted during daylight hours. The average time required for data collection was 7.4 minutes per segment.

### Data analysis

Intra-rater reliability (i.e. test-retest reliability) was evaluated by the consistency of judgments made by the same rater over a period of time, and inter-rater rater reliability was assessed based on the level of agreement achieved by independent raters. The intra-class correlation coefficients (ICCs) were calculated to assess reliability for continuous and ordinal variables. Agreement among dichotomous variables was assessed using Cohen’s Kappa statistic [25]. The adjectival ratings suggested by Landis and Koch [26] in the following categories were followed: 1.0 to 0.8 (almost perfect agreement), 0.8 to 0.6 (substantial agreement), 0.6 to 0.4 (moderate agreement), 0.4 to 0.2 (fair agreement), and 0.2 to 0.0 (poor agreement). The prevalence adjusted and bias adjusted Kappa (PABAK) coefficient was used to deal with items having high prevalence index or bias index [27]. For items with zero or little variance, the percentage of observed agreement of two raters was used to assess the reliability. The criterion for good level was set to no less than 75%, as used by Pikora and colleagues [19].

Construct validity refers to the extent to which an instrument measures what it claims to measure. Exploratory factor analysis (EFA) with equamax orthogonal rotation was applied to assess construct validity. Factor analysis appropriateness was assessed by Kaiser-Meyer-Olkin (KMO) value (greater than 0.7), and with a significant level less than 0.05 for Bartlett’s test of sphericity. Factor loadings with an absolute value greater than 0.40 were considered to be significant. The PASW version 18.0.0 (IBM Corporation, Somers, NY, USA) was used for data analysis.

## Results

### Inter-rater reliability

A total of 309 street segments (205 for the first scan and 104 for the second) were assessed to test inter-rater reliability. The results of inter-rater reliability are shown in Table 2 and Table 3. Seven items were not suitable for computing Kappa statistic or ICCs due to zero variance or data distribution characteristics; nevertheless, all of them had very high percentage of agreement (all > 85%). For items with adequate variance, the Kappa statistic was used to assess intra-rater reliability of 25 dichotomous items and the ICCs were used for 17 ordinal items. For Kappa values, eighteen out of 25 (72%) dichotomous items reached almost perfect level of agreement, and the rest 7 items (28%) also had a substantial level of agreement. Twelve out of 17 (71%) items using ICCs reached good level of agreement, and 3 items (22%) were in fair level. Two items, “Evenness and Bike lanes maintenance” and “Cigar butts or discarded cigarette”, showed poor reliability. All the six dimensions reached good level of inter-rater reliability (all ICCs > 0.75, p < 0.001). When the PABAK coefficient was applied, the inter-rater reliability showed varying degrees of improvement, especially for the items with higher prevalence index.

**Table 2:** Inter-rater and intra-rater reliability of the CUBEST

**Item**	**Inter-rater Reliability (n = 309)**					**Intra-rater Reliability (n = 208)**				
	**Agreement (%)**	**Kappa/ICCs**	**PABAK**	**Prevalence Index**	**Bias Index**	**Agreement (%)**	**Kappa/ICCs**	**PABAK**	**Prevalence Index**	**Bias Index**
***Residential Density***		0.853					0.563			
Row houses of 1–3 stories	100	NA				100	NA			
Apartment or condos 1–6 stories	94	0.776	0.871	0.65	0.02	83	0.367	0.663	0.69	0.06
Apartment or condos 7–12 stories	97	0.821	0.935	0.80	0.03	88	0.441	0.769	0.77	0.07
Apartments or condos more than 13 stories	95	0.811	0.909	0.72	0.01	89	0.500	0.788	0.76	0.01
***Street Connectivity***		0.902					0.721			
Number of intersections		0.961					0.798			
Presence of bus or other transit stops	99	0.992	0.990	0.38	0.01	96	0.891	0.913	0.45	0.04
Number of transit stops		0.972					0.786			
***Accessibility***		0.912					0.758			
Fast food restaurant	91	0.819	0.819	0.03	0.04	86	0.689	0.692	0.10	0.01
Other restaurant	88	0.718	0.767	0.42	0.01	81	0.522	0.625	0.47	0.04
Convenience or small grocery store	91	0.764	0.812	0.45	0.01	78	0.454	0.558	0.44	0.08
Supermarket	96	0.857	0.922	0.68	0.02	91	0.691	0.827	0.65	0.01
Shopping mall or Department store	93	0.733	0.851	0.67	0.04	83	0.414	0.663	0.66	0.09
Coffee shop or Tea house or Bar	94	0.809	0.877	0.60	0.01	86	0.528	0.711	0.63	0.05
Other retail stores	86	0.705	0.728	0.28	0	67	0.313	0.346	0.36	0.28
Other public or government service destinations	90	0.806	0.806	0.01	0.01	72	0.446	0.442	0.03	0.09
Other recreational destinations	95	0.830	0.909	0.68	0.03	79	0.313	0.576	0.63	0.12
Sidewalks/Bike lane	96	0.909	0.922	0.38	0.01	90	0.792	0.808	0.28	0.03
Indoor recreation or exercise facility	99	0.887	0.994	0.97	0.01	99	0.564	0.971	0.97	0.01
Outdoor sports/playing field	100	1.000				99	NA			
Public recreational equipments	99	0.916	0.974	0.83	0.01	96	0.744	0.923	0.84	0
School with recreational facilities	100	NA				100	NA			
Neighborhood public park	99	0.964	0.994	0.91	0.01	96	0.316	0.923	0.94	0.02
Municipal public park	99	0.897	0.987	0.94	0	100	1.000			
Public open space that is not a park	99	0.918	0.981	0.87	0.01	93	0.426	0.865	0.88	0.01
Lake or Beijing-Hangzhou Grand Canal	97	0.852	0.948	0.81	0.01	94	0.617	0.885	0.84	0.03
Parking lot or parking garage	86	0.712	0.715	0.11	0.05	73	0.459	0.461	0.14	0.13
Abandoned building or vacant lot	98	0.776	0.961	0.91	0.01	91	−0.044	0.817	0.91	0.02
Railroad, bridge, tunnel, or highway	97	0.852	0.948	0.81	0	92	0.537	0.846	0.82	0.01
***Walkability (segments with sidewalks)***		0.788					0.360			
Presence of sidewalks	98	0.959				90	0.894			
Sidewalk width	94	0.740				78	0.256			
Evenness and sidewalks maintenance	87	NA				83	NA			
Slope	99	NA				90	NA			
Obstructions on sidewalks	75	0.834				66	0.357			
Signs of “Avoid Pedestrians”/“Slow Down”	83	0.825				55	0.484			
***Bikeability (segments with bike lanes)***		0.766					0.587			
Presence of bike lane or marked shoulder	99	0.985				96	0.948			
Evenness and Bike lanes maintenance	96	0.308				93	0.910			
Slope	99	NA				99	NA			
Presence of speed limit signs on motor vehicle lanes	96	0.925	0.929	0.21	0.01	86	0.693	0.711	0.25	0.05
Street design to reduce volume or speed	84	0.882				67	0.744			
Crossing aids for pedestrians and bicyclists to cross the street safely	86	0.917				65	0.793			
Cars running or parked on bike lanes	74	0.757				58	0.468			
Cycling or walking against the flow of traffic on bike lanes	64	0.638				55	0.464			
***Aesthetic***		0.771					0.710			
Cultural landscape in the neighborhood	84	0.846				70	0.722			
Natural sights in the neighborhood	63	0.822				65	0.764			
Whole or broken liquor bottles or cans	100	NA				99	NA			
Cigar butts or discarded cigarette	76	0.391				64	0.314			
Garbage, litter, or broken glass	61	0.548				57	0.472			
Graffiti on the buildings, signs or walls	100	1.000				97	NA			
Unattended pets or lost animals	99	NA				96	NA			

**Table 3:** Summary of the inter-rater reliability of the CUBEST (N = 309)

**Dimension (Number of items)**	**ICCs for Dimensions**	**Kappa Statistic**						**ICCs**			
		**NA**^**a**^	**Slight (0–0.2)**	**Fair (0.2-0.4)**	**Moderate (0.4-0.6)**	**Substantial (0.6-0.8)**	**Almost Perfect (0.8-1.0)**	**NA**^**b**^	**Poor (0–0.4)**	**Fair (0.4-0.75)**	**Good (0.75-1.0)**
Residential Density (4)	0.853	1				1	2				
Street Connectivity (3)	0.902						1				2
Accessibility (21)	0.912	1				6	14				
Walkability (6)	0.788							2		1	3
Bikeability (8)	0.766						1	1	1	1	4
Aesthetics (7)	0.771							2	1	1	3
Total Number (49)		2				7	18	5	2	3	12

^a^2 items were not suitable for computing Kappa statistic due to zero variance or data distribution characteristic.

^b^5 items were not suitable for computing ICCs due to zero variance or data distribution characteristic.

### Intra-rater reliability (test-retest reliability)

A total of 208 street segments (104 segments for each rater) were evaluated to test intra-rater reliability. Table 2 and Table 4 indicate the results of intra-rater reliability for each item. The percentage of agreement was calculated for 9 items with zero variance or specific data distribution. And all of them had very high percentage of agreement (Evenness and sidewalks maintenance = 83%, rest 8 items > 90%). For the 24 dichotomous items with adequate variance, Kappa statistic was used to assess intra-rater reliability and ICCs were used for other 16 ordinal items. Nineteen out of 24 (79%) items using Kappa statistic reached at least moderate level of intra-rater reliability. Four items showed fair level and one item fell into the poor category (“Abandoned building or vacant lot”). ICCs for the 6 dimensions were: residential density (0.563), street connectivity (0.721), accessibility (0.758), sidewalk quality (0.360), bike lane quality (0.587), and aesthetic (0.710), all of which were statistically significant (all p < 0.001). After calculation of the PABAK coefficient, fifteen out of 23 items reached higher levels of intra-rater reliability.

**Table 4:** Summary of the intra-rater reliability of the CUBEST (N = 208)

**Dimension (Number of items)**	**ICCs for Dimensions**		**Kappa Statistic**					**ICCs**			
		**NA**^**a**^	**Slight (0–0.2)**	**Fair (0.2-0.4)**	**Moderate (0.4-0.6)**	**Substantial (0.6-0.8)**	**Almost Perfect (0.8-1.0)**	**NA**^**b**^	**Poor (0–0.4)**	**Fair (0.4-0.75)**	**Good (0.75-1.0)**
Residential Density (4)	0.563	1		1	2						
Street Connectivity (3)	0.721						1				2
Accessibility (21)	0.758	2	1	3	9	5	1				
Walkability (6)	0.360							2	2	1	1
Bikeability (8)	0.587						1	1		3	3
Aesthetics (7)	0.710							3	1	2	1
Total Number (49)		3	1	4	11	5	3	6	3	6	7

^a^3 items were not suitable for computing Kappa statistic due to zero variance or data distribution characteristic.

^b^6 items were not suitable for computing ICCs due to zero variance or data distribution characteristic.

### Construct validity

The KMO value in the present analysis was 0.758, which was within the range of “acceptable” values. The Bartlett’s test of sphericity was 1593 and significant at p < 0.001, which indicated a highly significant correlation among the survey questions. This information allowed us to identify the factor model using the EFA approach.

The EFA yielded 6 factors with eigenvalues greater than 1, which explained 60.0% of the variance. After suppressing small coefficients with absolute value below 0.40, the rotated component matrix is shown in Table 5. Almost perfect matching was found between 6 components and the corresponding items designed for them, which suggests very good construct validity of the CUBEST. But still, the main factor loadings of a few items (or summary scores of items) deviated from their original design. These were “Presence of bus or other transit stops” designed for street connectivity, “Evenness and sidewalks maintenance” designed for sidewalk quality, “Crossing aids for pedestrians and bicyclists” designed for bike lane quality, and “Cultural features” and “Nature sights” designed for aesthetic.

**Table 5:** **Construct Validity of the CUBEST**^**a**^**: rotated component matrix using factor analysis**

	**Residential Density**	**Street Connectivity**	**Accessibility**	**Sidewalk quality**	**Bike lane quality**	**Aesthetics**
Residential Density^b^	*0.893*					
Number of Intersections		*0.741*				
Presence of bus or other transit stops		0.475			*0.542*	
Commercial destinations^b^		*0.583*				
Physical activity destinations^b^			*0.703*			
Parking lot or parking garage			*−0.417*			
Railroad, bridge, tunnel, or highway			*0.565*			
Presence of sidewalks				*0.558*		
Sidewalk width				*0.556*		
Evenness and sidewalks maintenance						*0.781*
Obstructions on sidewalks				*0.498*		0.481
Signs of “Avoid Pedestrians”/“Slow Down”				*−0.568*		
Presence of bike lanes					*0.911*	
Presence of speed limit signs on motor vehicle lanes					*−0.509*	
Street design characteristics to reduce volume or speed		0.558			*0.566*	
Crossing aids for pedestrians and bicyclists to cross the street safely		*0.602*			0.588	
Cars running or parked on bike lanes					*−0.832*	
Cycling or walking against the flow of traffic on bike lanes					*−0.841*	
Cultural features in the neighborhood		0.418	*0.576*			
Natural sights in the neighborhood			*0.791*			
Cigar butts or discarded cigarette						*0.733*
Garbage, litter, or broken glass						*0.765*
Graffiti on the buildings, signs or walls				−0.492		*0.455*

^a^After suppressing small coefficients (absolute value below 0.40).

^b^Using total score or summary score to represent original items.

### Final Scan Tool

Six dimensions and 41 items were included in the final CUBEST specific to Hangzhou City (Q1 to Q41), as shown in Table 6. Items with zero or little variance were excluded because of little distinction among characteristics of street segments in Hangzhou, which includes “Abandoned building or vacant lot”, “Slope of sidewalks”, “Slope of bike lanes”, “Whole or broken liquor bottles or cans”, and “Unattended pets or lost animals”. However, these items should be viewed as back-up items and added when applying the CUBEST to other Chinese cities. For example, slope of sidewalks and bike lanes should be added when assessing the built environment in cites with hilly terrain, like Qingdao and Chongqing.

**Table 6:** Dimensions and items included in the final CUBEST specific to Hangzhou City

**Dimensions**					
**Residential density**	**Street connectivity**	**Accessibility**	**Sidewalk quality**	**Bike lane quality**	**Aesthetic**
Types of Housing ^a^ (Q1-Q4)	Number of intersections (Q5)	Commercial destinations ^b^ (Q8-Q14)	Presence of sidewalks (Q25)	Presence of bike lanes (Q30)	Cultural features (Q37)
	Presence of bus or other transit stops (Q6)	Physical activity destinations ^c^ (Q15-Q22)	Sidewalk width (Q26)	Evenness and maintenance (Q31)	Natural sights (Q38)
	Number of transit stops (Q7)	Other types of destinations ^d^ (Q23-Q24)	Evenness and maintenance (Q27)	Presence of speed limit signs on motor vehicle lanes (Q32)	Cigar butts or discarded cigarette (Q39)
			Obstructions (Q28)	Street design characteristics to reduce volume or speed (Q33)	Garbage, litter, or broken glass (Q40)
			Signs of “Avoid Pedestrians”/“Slow Down” (Q29)	Crossing aids for pedestrians and bicyclists to cross the street safely (Q34)	Graffiti on the buildings, signs or walls (Q41)
				Cars running or parked on bike lanes (Q35)	
				Cycling or walking against the flow of traffic on bike lanes (Q36)	

^a^Row houses; apartment of 1–6, 7–12, more than 13 stories.

^b^Fast food; other restaurant; convenience/grocery store; coffee shop or tea house; other retail stores; other service destinations; other recreational destinations.

^c^Sidewalks / Bike lane; indoor fitness venues; outdoor sports field; school with facilities; neighborhood park; municipal park; public open space; lake or canal.

^d^Parking lot; Railroad, bridge, tunnel, or highway.

## Discussion

In this study, we developed the instrument designed specifically for measuring the urban built environment related to physical activity in the Chinese context and conducted necessary reliability and validity tests. In general, most of the items demonstrated at least substantial level of inter-rater reliability (Kappa > 0.6, ICCs > 0.75), as well as fair to acceptable levels of agreement for the intra-rater reliability. Of the 6 dimensions, the accessibility dimension showed the highest reliability, which was consistent with Brownson’s study [13].

A low ICC or Kappa statistic could be attributed to actual low reliability, a change of the environment between the first and the second audit, the subjectivity of judgments, and/or little variation across segments [28]. Items measuring access to destinations changed little over time, which ensured higher intra-rater reliability. By contrast, some items measuring aesthetic, sidewalk, and bike lane quality varied over time (e.g. “Obstructions on bike lanes”, or “Cigar butts or discarded cigarette”), which resulted in lower intra-rater reliability. To minimize the variation in street environment over time, a two-week time interval between the first and second audit was chosen, which also providing sufficient washout period to avoid memory effect. In addition, necessary subjective judgment for a few items can lead to lower inter-rater reliability despite of proper auditor training [9,22]. These items included obstacles on sidewalks and bike lanes and aesthetic items.

Little variance or certain data distribution can also cause problems (e.g. lower Kappa statistic due to high prevalence effect). The PABAK coefficient was reported alongside of the original Kappa coefficient, which was especially necessary when the prevalence index or the bias index was high. The item “Presence of community park” presented perfect observer agreement (96%), however, the original Kappa coefficient was only 0.316 due to high prevalence index. The PABAK adjusted for prevalence effect reached 0.923, much higher than unadjusted value. Adjustments for prevalence and bias effect increased the Kappa coefficient in 4 items in the inter-rater reliability test and 15 items in the intra-rater reliability test. Hoehler [29] is critical of the use of the PABAK because the effects of bias and prevalence on the magnitude of kappa are themselves informative and should not be adjusted for. Alongside the obtained values of Kappa and the PABAK, we also reported the prevalence index and bias index to provide more valuable information as recommended by other authors [27,30].

Factor analysis was used to test construct validity. The six extracted principal components were basically in accordance with the theoretical framework of the CUBEST. A few items showed larger factor loading on dimensions to which they did not belong, however, these results can be explained. For example, the commercial buildings usually were located at places where streets are well-connected to increase business visibility. The analysis correspondingly indicated that the commercial destination items loaded highly on the factor of street connectivity instead of accessibility. Similarly, “Evenness and sidewalks maintenance” could not only be used to measure the sidewalk quality, but also be in reflection of the aesthetic quality. Another example is that, the “Presence of bus or other transit stops” loaded strongly on bike lane quality instead of street connectivity because the public bicycle rental stations are usually built near bus stops to facilitate the traffic movement.

A 91-item Environment in Asia Scan Tool – Hong Kong (EAST_HK) was developed by Cerin et al. to objectively assess aspects of the neighborhood environment hypothesized to affect walking in Hong Kong and similar ultra-dense Asian metropolises [9]. There were differences in several aspects between the CUBEST and EAST_HK. First, items hypothesized to affect cycling behavior were added in the CUBEST given that cycling remains one of the most popular form of transport in China. Second, the EAST_HK included 41 items as a list of destinations. However, we had special focus of the CUBEST on two most important types of destinations, that was commercial (8 items) and physical activity destinations (8 items), and had other types of destinations simplified to keep the questionnaire as short as possible. A simplified instrument would be a more feasible option for large-scale survey. Third, items measuring safety from crime were not included in the CUBEST due to consideration of hard judgment for raters during a short stop in the street. In addition, additional items were added, like the presence and number of transit stops to better capture variation of street connectivity.

We acknowledge the limitations of this study. One is the lack of criterion validity test for the instrument. GIS data could be used as criterion measure to test the validity of residential density, street connectivity, and land-use mix. Unfortunately, poor accessibility and high cost make the use of GIS data impossible in this and many other studies conducted in China [31]. The other limitation is that this study took place in one city of China, which may limit the application of the CUBEST to other Chinese cities. However, we evaluated the CUBEST in three different types of administrative planning units, which to some extent ensured enough variations in the environmental features. When applying the CUBEST in other Chinese cities, we suggest that some items should be added or modified to capture specific built environment features to different cities.

## Conclusions

The CUBEST is a reliable and valid instrument that can be used to assess the physical activity-related built environment in Hangzhou, and potentially other cities in China (with suitable modifications).

## Competing interests

The authors declare no conflict of interests.

## Authors’ contributions

MS participated in the development of the CUBEST, performed the statistical analysis and drafted the manuscript. YKD participated in the development of the CUBEST and collected the data. QML and YJR participated in the study coordination. IK helped to draft the manuscript. JL and LML conceived of the study and participated in its design. All authors read and approved the final manuscript.

## Pre-publication history

The pre-publication history for this paper can be accessed here:

http://www.biomedcentral.com/1471-2458/14/109/prepub
